# Supplementary material for: Hepatocyte-specific loss of GPS2 in mice reduces non-alcoholic steatohepatitis via activation of PPARα
Source: Nat Commun. 2019 Apr 11;10:1684. doi: 10.1038/s41467-019-09524-z (PMC6459876; doi:10.1038/s41467-019-09524-z)
Supplement: Supplementary file 1 — n [file 41467_2019_9524_MOESM1_ESM.pdf]

Hepatocyte-specific loss of GPS2 in mice reduces non-alcoholic steatohepatitis via activation of PPARα.

Liang et al.

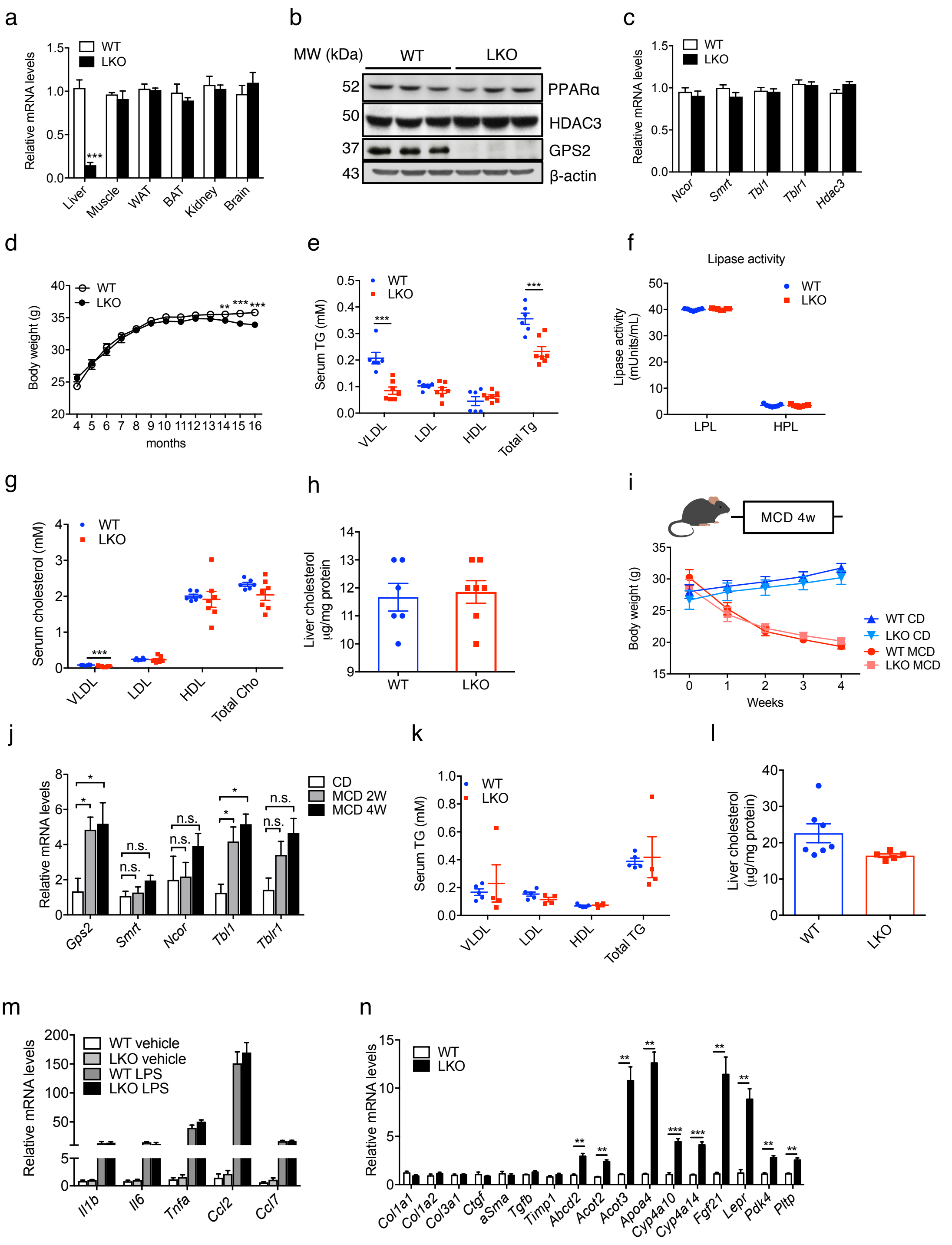

a
Relative mRNA levels
WT
LKO
Liver
Muscle
WAT
BAT
Kidney
Brain
b
MW (kDa)
WT
LKO
PPARα
HDAC3
GPS2
β-actin
c
Ncor
Smrt
Tbl1
Tblr1
Hdac3
d
Body weight (g)
months
e
Serum TG (mM)
VLDL
LDL
HDL
Total Tg
f
Lipase activity
Lipase activity (mUnits/mL)
LPL
HPL
g
Serum cholesterol (mM)
Total Cho
h
Liver cholesterol µg/mg protein
i
MCD 4w
Body weight (g)
WT CD
LKO CD
WT MCD
LKO MCD
Weeks
j
CD
MCD 2W
MCD 4W
Gps2
n.s.
k
Total TG
l
Liver cholesterol (µg/mg protein)
m
WT vehicle
LKO vehicle
WT LPS
LKO LPS
Il1b
Il6
Tnfa
Ccl2
Ccl7
n
Col1a1
Col1a2
Col3a1
Ctgf
aSma
Tgfb
Timp1
Abcd2
Acot2
Acot3
Apoa4
Cyp4a10
Cyp4a14
Fgf21
Lepr
Pdk4
Pltp

Supplementary Figure 1: Phenotyping of *Gps2* LKO mice.

(a) QPCR analysis of *Gps2* expression in different organs in CD fed WT and LKO mice, n = 3, non-parametric Mann-Whitney test. (b) Western blot analysis of PPARα, HDAC3 and GPS2 in WT and LKO mice, β-actin was used as an internal control. (c) QPCR analysis of corepressor complex components in CD fed WT and LKO mice, n = 3, non-parametric Mann-Whitney test. (d) Body weight change in CD fed mice, WT n = 6-12, LKO n = 6-11, non-parametric Mann-Whitney test; (e) serum lipoprotein, WT n = 6, LKO n = 7, non-parametric Mann-Whitney test; and (f) plasma lipase activity, n = 7, non-parametric Mann-Whitney test; (g) serum and (h) liver cholesterol in 6 hour fasted WT and LKO mice, WT n = 6 and LKO n = 7, non-parametric Mann-Whitney test. (i) Body weight change of MCD fed mice, n = 4 in CD and n = 10-12 in MCD groups. (j) QPCR analysis of corepressor complex component expression in different time points of MCD fed mice, n = 3 in CD, n = 6 in 2 weeks of MCD and n = 4 in 4 weeks of MCD, one-way ANOVA followed by Tukey's test. (k) Serum and (l) liver cholesterol in 6 hour fasted WT and LKO mice after 4 weeks of MCD, WT n = 5 and LKO n = 4, non-parametric Mann-Whitney test. (m-n) QPCR analysis of mice liver mRNA expression in (l) LPS treatment; or (m) basal condition, vehicle n = 5 and LPS n = 4, non-parametric Mann-Whitney test. All data are represented as mean ± s.e.m. *P < 0.05, **P < 0.01, ***P < 0.001.

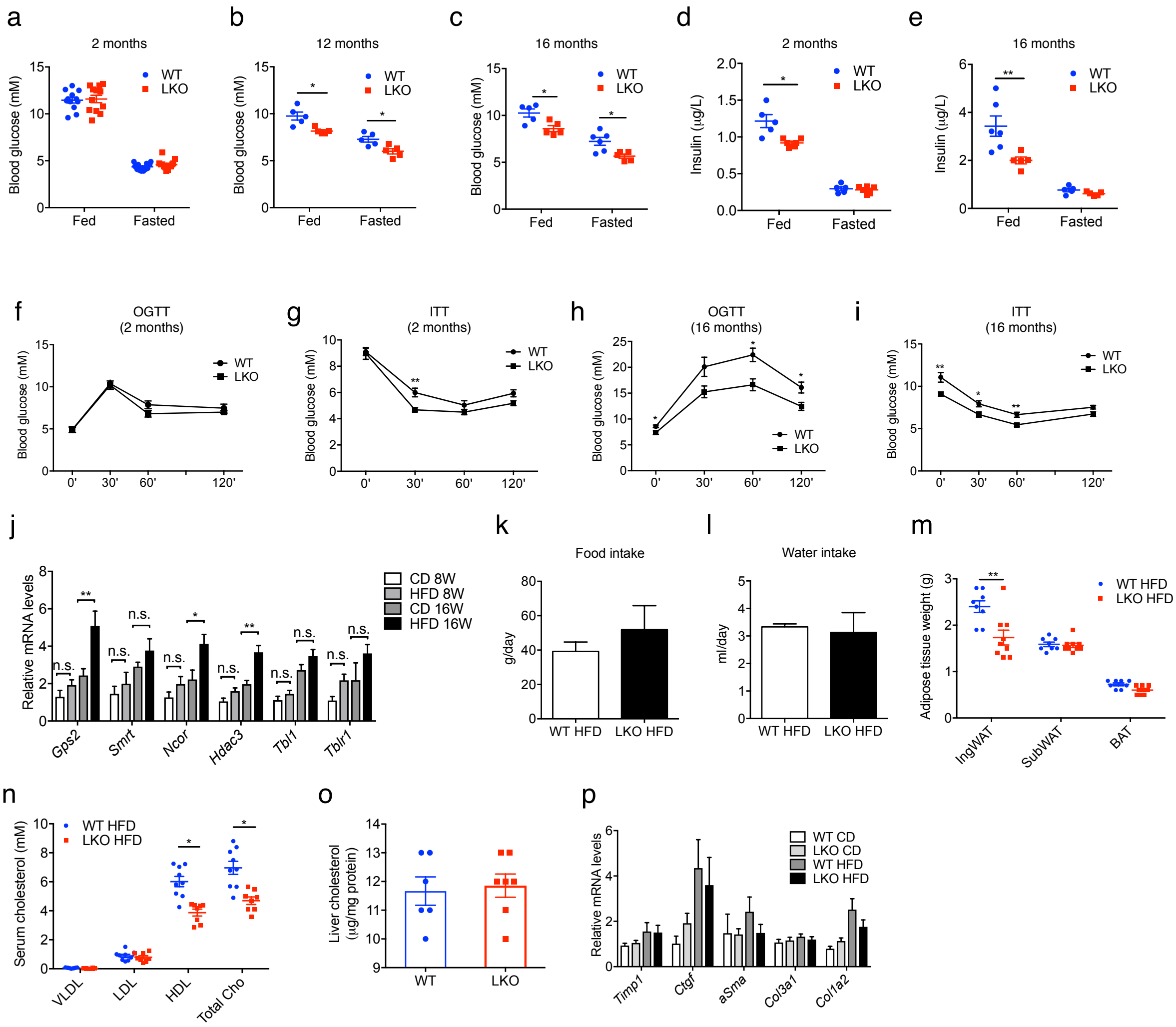

a
2 months
Blood glucose (mM)
WT
LKO
Fed
Fasted
b
12 months
c
16 months
d
2 months
Insulin (μg/L)
e
16 months
f
OGTT
(2 months)
g
ITT
(2 months)
h
OGTT
(16 months)
i
ITT
(16 months)
j
Relative mRNA levels
CD 8W
HFD 8W
CD 16W
HFD 16W
Gps2
Smrt
Ncor
Hdac3
Tbl1
Tblr1
k
Food intake
g/day
WT HFD
LKO HFD
l
Water intake
ml/day
m
Adipose tissue weight (g)
IngWAT
SubWAT
BAT
n
Serum cholesterol (mM)
VLDL
LDL
HDL
Total Cho
o
Liver cholesterol
(μg/mg protein)
WT
LKO
p
Relative mRNA levels
WT CD
LKO CD
WT HFD
LKO HFD
Timp1
Ctgf
aSma
Col3a1
Col1a2

Supplementary Figure 2: Phenotyping of *Gps2* LKO mice.

(a-e) Fed and 12 hour fasted blood glucose in (a) 2 months old, n = 12 (b) 12 months old, n = 5; and (c) 16 months old mice, n = 5-6. (d-e) Fed and 12 hour fasted insulin in (d) 2 months old, WT n = 5 and LKO n = 6; and (e) 16 months old mice, WT n = 6 and LKO n = 5. (f-i) (f) OGTT, n = 6; and (g) ITT in 2 months old mice, n = 5. (h-i) (h) OGTT and (i) ITT in 16 months old mice, non-parametric Mann-Whitney test. (j) QPCR analysis of corepressor complex components in 8 weeks and 16 weeks of CD or HFD fed mice, n = 5 in each group, one-way ANOVA followed by Tukey's test. (k-l) (k) Food and (l) water uptake of the mice after 12 weeks of HFD feeding, n = 9. (m) Adipose tissue weight in HFD fed mice, WT n = 8 and LKO n = 9. (n) 6 hour fasted serum lipoprotein cholesterol profiles and (o) liver cholesterol in WT and LKO mice after 12 weeks of HFD, WT n = 6 and LKO n = 7, non-parametric Mann-Whitney test. (p) QPCR analysis of fibrotic genes in CD and HFD fed WT and LKO mice, n = 5, one-way ANOVA followed by Tukey's test. All data are represented as mean ± s.e.m. *P < 0.05, **P < 0.01, ***P < 0.001.

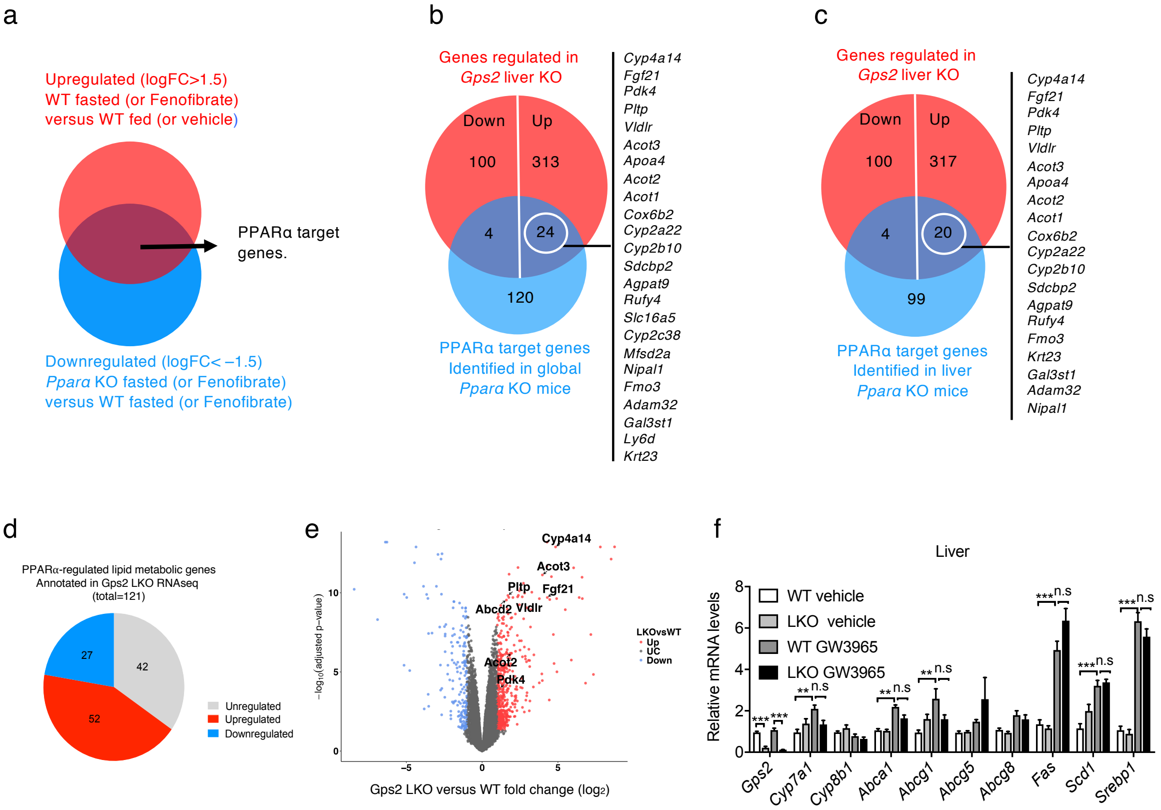

a
Upregulated (logFC>1.5)
WT fasted (or Fenofibrate)
versus WT fed (or vehicle)
PPARα target
genes.
Downregulated (logFC< −1.5)
Ppara KO fasted (or Fenofibrate)
versus WT fasted (or Fenofibrate)
b
Genes regulated in
Gps2 liver KO
Down
Up
100
313
4
24
120
PPARα target genes
Identified in global
Ppara KO mice
Cyp4a14
Fgf21
Pdk4
Pltp
Vldlr
Acot3
Apoa4
Acot2
Acot1
Cox6b2
Cyp2a22
Cyp2b10
Sdcbp2
Agpat9
Rufy4
Slc16a5
Cyp2c38
Mfsd2a
Nipal1
Fmo3
Adam32
Gal3st1
Ly6d
Krt23
c
Genes regulated in
Gps2 liver KO
Down
Up
100
317
4
20
99
PPARα target genes
Identified in liver
Ppara KO mice
Cyp4a14
Fgf21
Pdk4
Pltp
Vldlr
Acot3
Apoa4
Acot2
Acot1
Cox6b2
Cyp2a22
Cyp2b10
Sdcbp2
Agpat9
Rufy4
Fmo3
Krt23
Gal3st1
Adam32
Nipal1
d
PPARα-regulated lipid metabolic genes
Annotated in Gps2 LKO RNAseq
(total=121)
27
42
52
Unregulated
Upregulated
Downregulated
e
Cyp4a14
Acot3
Pltp
Fgf21
Abcd2
Vldlr
Acot2
Pdk4
LKOvsWT
Up
UC
Down
−log10(adjusted p−value)
Gps2 LKO versus WT fold change (log2)
f
Liver
WT vehicle
LKO vehicle
WT GW3965
LKO GW3965
Relative mRNA levels
Gps2
Cyp7a1
Cyp8b1
Abca1
Abcg1
Abcg5
Abcg8
Fas
Scd1
Srebp1
n.s

Supplementary Figure 3: GPS2 interplays with PPARα in mice liver.

(a) Flowchart representing the comparative analysis of transcriptomic data using different datasets. (b-c) Comparative analysis of *Gps2* LKO and (b) *Ppara* KO and (c) *Ppara* liver-specific KO transcriptomes. (d) Pie chart representing the percentage of GPS2 regulated genes among the 121 PPARα target metabolic genes. (e) Volcano plot of the global mRNA expression in LKO liver. (f) QPCR analysis of WT and LKO livers in vehicle and GW3965 (LXR agonist) treated mice, vehicle n = 3-7, GW3965 n = 6, one-way ANOVA followed by Tukey's test. All data are represented as mean ± s.e.m. *P < 0.05, **P < 0.01, ***P < 0.001.

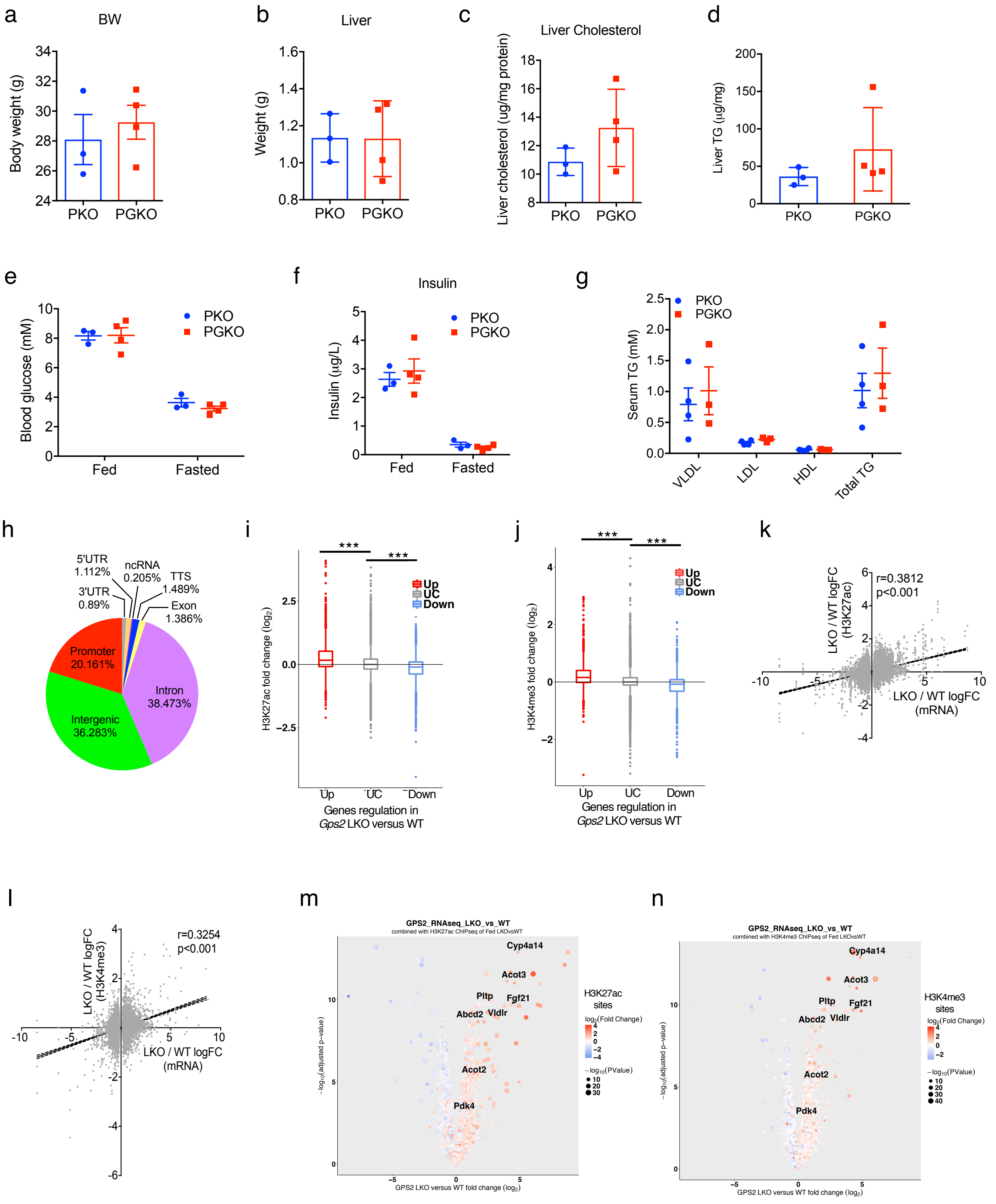

a
BW
Body weight (g)
PKO
PGKO
b
Liver
Weight (g)
PKO
PGKO
c
Liver Cholesterol
Liver cholesterol (ug/mg protein)
PKO
PGKO
d
Liver TG (µg/mg)
PKO
PGKO
e
Blood glucose (mM)
PKO
PGKO
Fed
Fasted
f
Insulin
Insulin (µg/L)
PKO
PGKO
Fed
Fasted
g
Serum TG (mM)
PKO
PGKO
VLDL
LDL
HDL
Total TG
h
5'UTR 1.112%
ncRNA 0.205%
TTS 1.489%
3'UTR 0.89%
Exon 1.386%
Promoter 20.161%
Intron 38.473%
Intergenic 36.283%
i
H3K27ac fold change (log2)
Up
UC
Down
Genes regulation in Gps2 LKO versus WT
j
H3K4me3 fold change (log2)
Up
UC
Down
Genes regulation in Gps2 LKO versus WT
k
LKO / WT logFC (H3K27ac)
r=0.3812
p<0.001
LKO / WT logFC (mRNA)
l
LKO / WT logFC (H3K4me3)
r=0.3254
p<0.001
LKO / WT logFC (mRNA)
m
GPS2_RNAseq_LKO_vs_WT
Cyp4a14
Acot3
Pltp
Fgf21
Abcd2
Vldlr
Acot2
Pdk4
H3K27ac sites
GPS2 LKO versus WT fold change (log2)
n
GPS2_RNAseq_LKO_vs_WT
Cyp4a14
Acot3
Pltp
Fgf21
Abcd2
Vldlr
Acot2
Pdk4
H3K4me3 sites
GPS2 LKO versus WT fold change (log2)

Supplementary Figure 4: Further characterization of the GPS2-PPARa relationship in mice.

(a) Body weight; (b) liver weight; (c) 6 hour fasted liver cholesterol; (d) liver triglyceride; (e) fed and 12 hour fasted blood glucose; (f) insulin and (g) 6 hour fasted serum lipoprotein triglyceride in PKO and PGKO mice, PKO n = 3 and PGKO n = 4. (h) Global analysis of GPS2 peak distributions. Average logFC at (i) H3K27ac and (j) H3K4me3 sites in *Gps2* LKO versus WT of transcriptional (RNAseq) upregulated, downregulated and unchanged (UC) genes. (k-l) Global correlation analysis of mRNA logFC with (k) H3K27ac site logFC; and (l) H3K4me3 site logFC in LKO versus WT mice, nonparametric Spearman's test. (m-n) Volcano plot representing global gene expression along with logFC of (m) H3K27ac and (n) H3K4me3 sites (color from blue to red) and -log(P-value) (dot size) in LKO versus WT livers. Data are represented as mean ± s.e.m. All the comparisons were done by two-tailed, non-parametric Mann-Whitney test. $^{*}P < 0.05$, $^{**}P < 0.01$, $^{***}P < 0.001$.

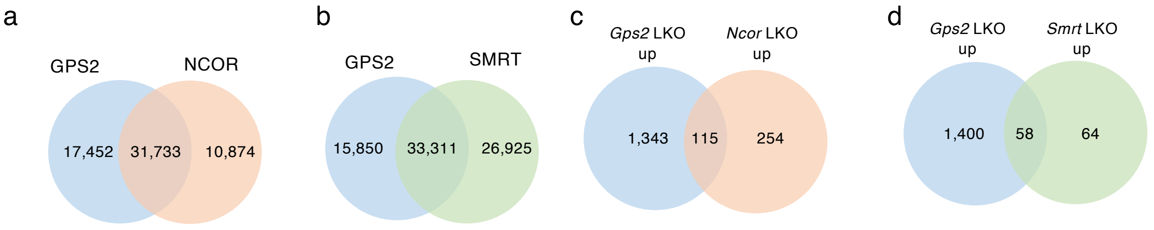

e

Pathway	Gene	Statistics (adj. P value)
Metabolic pathways	23	5.78E-15
Retinol metabolism	10	1.04E-14
Drug metabolism - cytochrome P450	10	2.50E-14
PPAR signaling pathway	8	4.03E-11

f

Pathway	Gene	Statistics (adj. P value)
Prion diseases	3	3.21E-05
Chagas disease (American trypanosomiasis)	4	3.21E-05
Progesterone-mediated oocyte maturation	4	3.21E-05
Oocyte meiosis	4	3.21E-05

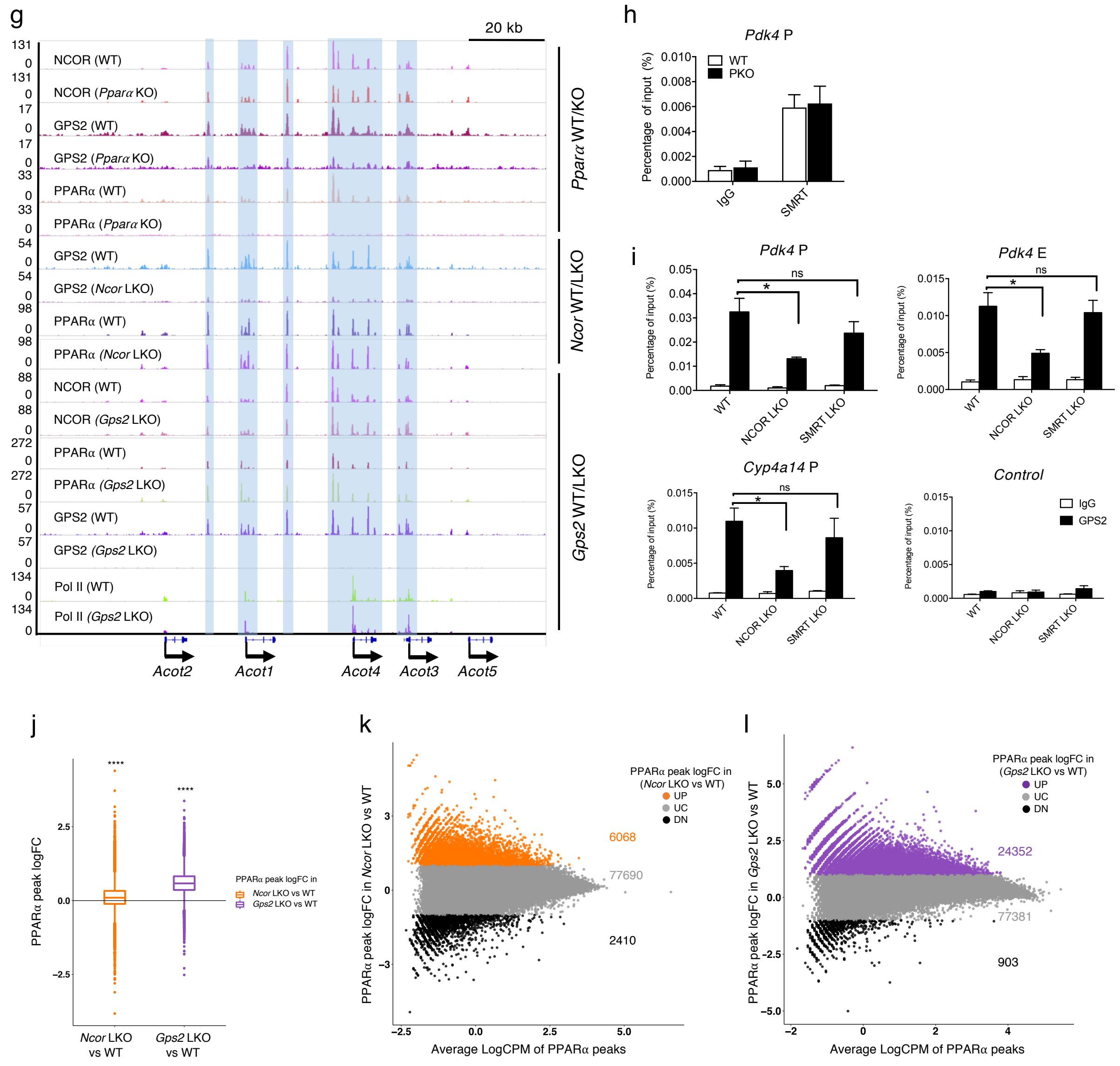

Supplementary Figure 5: Comparative analysis of GPS2, NCOR and SMRT transcriptome and cistrome.

(a-b) Comparative analysis of GPS2, (a) NCOR or (b) SMRT binding sites in mouse liver. (c-d) Comparative analysis of *Gps2* and (c) *Ncor* LKO and (d) *Smrt* LKO transcriptome data. (e-f) KEGG analysis of (e) GPS2/NCOR; and (f) GPS2/SMRT coregulated genes. (g) ChIP/seq tracks representing NCOR, GPS2, PPARα recruitment in *Ppara*, *Ncor*, *Gps2* KO and respective WT livers in *Acot1-5* loci. (h) ChIP qPCR analysis of SMRT recruitment in WT and PKO livers, non-parametric Mann-Whitney test. (i) ChIP qPCR analysis of GPS2 recruitment in WT, *Ncor* and *Smrt* KO livers in *Pdk4* promoter (upper left), enhancer (upper right), *Cyp4a14* promoter (lower left) and control regions (lower right), WT n = 6, *Ncor* KO n = 3 and *Smrt* KO n = 3. (j) Box plot representing average logFC of PPARα peaks in *Ncor* LKO and *Gps2* LKO versus WT livers, n = 3 in each group. (k-l) Smear plot of average logFC of PPARα peaks in (k) *Ncor* LKO versus WT; and (l) *Gps2* LKO versus WT livers, against average logCPM, n = 3 in each group. One-way ANOVA, followed by Tukey's test. All data are represented as mean ± s.e.m. *P < 0.05, **P < 0.01, ***P < 0.001.

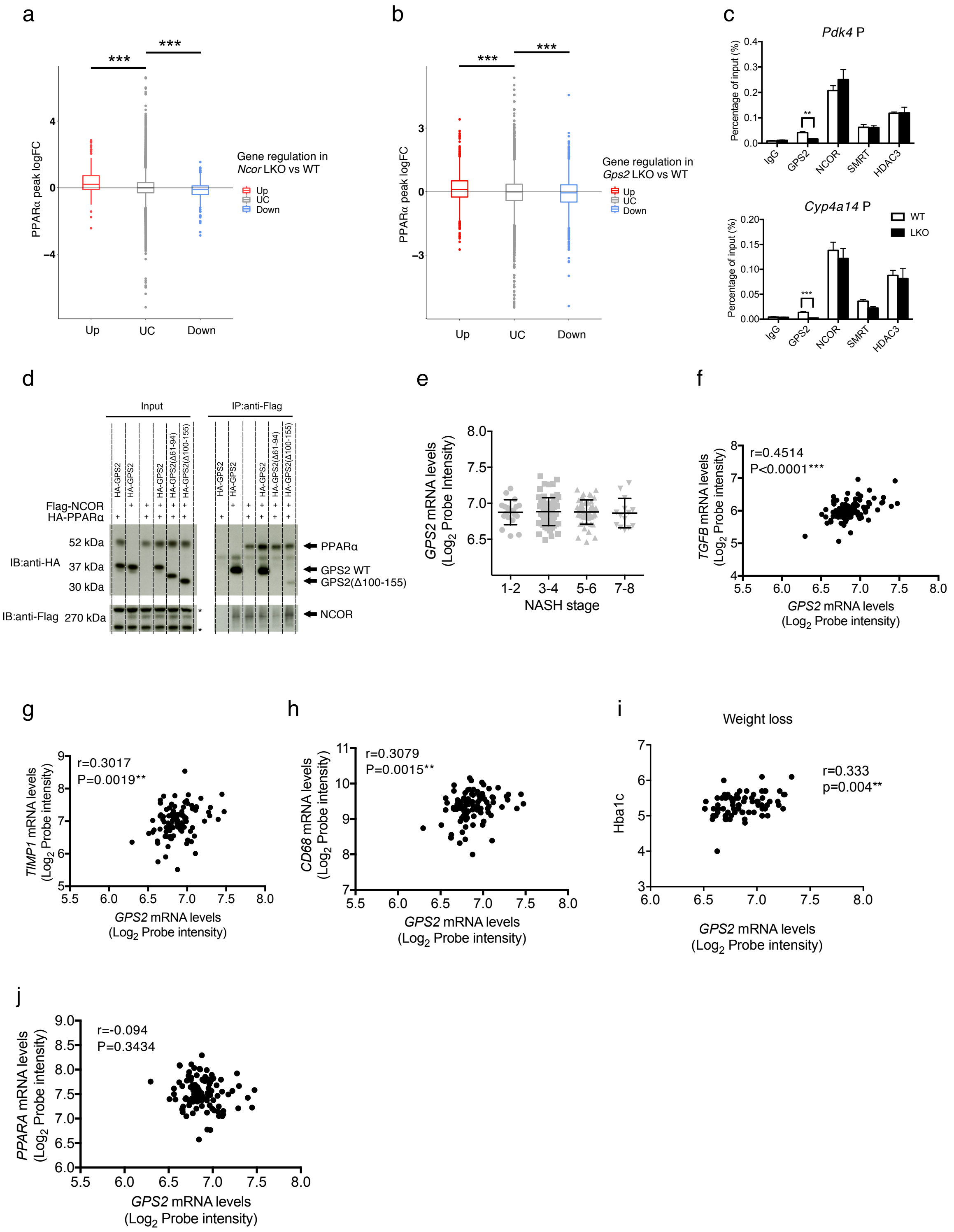
a
***
***
PPARα peak logFC
4
0
−4
Up
UC
Down
Gene regulation in
Ncor LKO vs WT
Up
UC
Down
b
***
***
PPARα peak logFC
3
0
−3
Up
UC
Down
Gene regulation in
Gps2 LKO vs WT
Up
UC
Down
c
Pdk4 P
Percentage of input (%)
0.4
0.3
0.2
0.1
0.0
**
IgG
GPS2
NCOR
SMRT
HDAC3
Cyp4a14 P
WT
LKO
Percentage of input (%)
0.20
0.15
0.10
0.05
0.00
***
IgG
GPS2
NCOR
SMRT
HDAC3
d
Input
IP:anti-Flag
HA-GPS2
HA-GPS2
HA-GPS2
HA-GPS2(Δ61-94)
HA-GPS2(Δ100-155)
HA-GPS2
HA-GPS2
HA-GPS2
HA-GPS2(Δ61-94)
HA-GPS2(Δ100-155)
Flag-NCOR
HA-PPARα
52 kDa
IB:anti-HA
37 kDa
30 kDa
IB:anti-Flag
270 kDa
PPARα
GPS2 WT
GPS2(Δ100-155)
NCOR
e
GPS2 mRNA levels
(Log2 Probe Intensity)
8.0
7.5
7.0
6.5
1-2
3-4
5-6
7-8
NASH stage
f
TGFB mRNA levels
(Log2 Probe intensity)
r=0.4514
P<0.0001***
8
7
6
5
4
5.5
6.0
6.5
7.0
7.5
8.0
GPS2 mRNA levels
(Log2 Probe intensity)
g
TIMP1 mRNA levels
(Log2 Probe intensity)
r=0.3017
P=0.0019**
9
8
7
6
5
5.5
6.0
6.5
7.0
7.5
8.0
GPS2 mRNA levels
(Log2 Probe intensity)
h
CD68 mRNA levels
(Log2 Probe intensity)
r=0.3079
P=0.0015**
11
10
9
8
7
5.5
6.0
6.5
7.0
7.5
8.0
GPS2 mRNA levels
(Log2 Probe intensity)
i
Weight loss
Hba1c
r=0.333
p=0.004**
7
6
5
4
3
6.0
6.5
7.0
7.5
8.0
GPS2 mRNA levels
(Log2 Probe intensity)
j
PPARA mRNA levels
(Log2 Probe intensity)
r=-0.094
P=0.3434
9.0
8.5
8.0
7.5
7.0
6.5
6.0
5.5
6.0
6.5
7.0
7.5
8.0
GPS2 mRNA levels
(Log2 Probe intensity)

Supplementary Figure 6: *GPS2* mRNA correlation analysis in NASH and fibrosis human livers.

(a-b) Average logFC of PPARα recruitment in (a) *Ncor* LKO versus WT at NCOR regulated genes (up- or down-regulated in *Ncor* LKO versus WT mice); and (b) *Gps2* LKO versus WT at GPS2 regulated genes (up- or down-regulated in *Gps2* LKO versus WT mice). One-way ANOVA followed by Tukey's test. (c) ChIP qPCR analysis of GPS2, NCOR, SMRT and HDAC3 recruitment in *Pdk4* (upper panel) and *Cyp4a14* (lower panel) promoters in WT and LKO mice livers, WT n = 4-12 and LKO n = 4-10, non-parametric Mann-Whitney test. (d) Co-immunoprecipitation of PPARα with NCOR in WT or Δ61-94 and Δ100-155 truncated GPS2 co-transfected 293 cells (* represents non-specific bands). (e) Liver expression of GPS2 in different NASH stages in human NASH dataset (GSE83452), n = 104, one-way ANOVA followed by Tukey's test. (f-j) Correlative analysis of *GPS2* with (f) *TGFB*, (g) *TIMP1*, (h) *CD68* and (i) HbA1c in human NASH liver dataset (GSE83452), n = 104, non-parametric Spearman's test. (j) Correlation of GPS2 with *PPARA* in human NAFLD liver samples (GSE83452), n = 104, non-parametric Spearman's test $^*P < 0.05$, $^{**}P < 0.01$, $^{***}P < 0.001$.

Gene	Forward (5'-3')	Reverse (5'-3')
36b4	ACTGGTCTAGGACCCGAGAAG	TCCCACCTTGTCTCCAGTCT
Abca1	CCCAGAGCAAAAAGCGACTC	GGTCATCATCACTTTGGTCCTTG
Abcd2	CATTATCACTGCAACGGGCTTT	TCCGATCGCTAACCATAGCCT
Abcg1	CAAGACCCTTTTGAAAGGGATCTC	GCCAGAATATTCATGAGTGTGGAC
Abcg5	CCTGCTGAGGCGAGTAACAA	GGACGCGGAGAAGGTAGAAA
Abcg8	CCTGTGGATAGTGCCTGCAT	GGAGAAGGTGAAGTTGCCGA
Acot2	AGTGCCTATGAAGGACTGAGGA	GGCAGAAAGCACCTTTACCA
Acot3	CAGTCACCCTCAGGTAACAGG	AAGTTTCCGCCGATGTTGGA
Apoa4	GGCCAATGTGGTGTGGGATT	TTGTCCTGGAAGAGGGTACTGA
aSma	CAGTCGCTGTCAGGAACCCT	GATGGATGGGAAAACAGCCCT
Ccl2	CAGATGCAGTTAACGCCCCA	TGAGCTTGGTGACAAAAACTACAG
Ccl7	GATCTCTGCCACGCTTCTGT	TGTCTTGAAGATAACAGCTTCCCA
Col1a1	CACCCTCAAGAGCCTGAGTC	TCGATCCAGTACTCTCCGCT
Col1a2	AGGAAAGAGAGGGTCTCCCG	GCCAGGAGGACCCATTACAC
Col3a1	GTCCAGGGATACGGGGTATG	CAGGGAAACCCATGACACCA
Ctgf	AGAACTGTGTACGGAGCGTG	GTGCACCATCTTTGGCAGTG
Cyp4a10	GAACTTCCCAAGTGCCTTTCC	CCTTTGGATCTGATCGCCCC
Cyp4a14	TCGGGGAGCAATATACGAGTCC	GGAGCAAACCATAACCAATCCAG
Cyp7a1	GGTCTGCCTGGAAAGCACTA	AGCATCGAAGATTTCCGGGT
Cyp8b1	TTGCAAATGCTGCCTCAACC	TAACAGTCGCACACATGGCT
Fasn	TGGGTGTGGAAGTTCGTCAG	CTGTCGTGTCAGTAGCCGAG
Fgf21	CTACCAAGCATA CCCCATCC	GCCTACCACTGTTCCATCCT
Gps2	GAAGCACCAGCTTTTCTTGCAGC	GCACTTGTGGTCCAAACATCTGC
Hdac3	TACAGCAGGCCAGAAGCACCCA	TGGGGAAACCATACTTTCCTTCCCA
Il1b	AAATACCTGTGGCCTTGGGC	CTTGGGATCCACACTCTCCAG
Il6	GCTGGAGTCACAGAAGGAGTGGC	TCTGACCACAGTGAGGAATGTCCA
Lepr	CGCCAGCTAGGTGTAAACTGG	GAAGGGGTTCTTAGGTAATGGC
Ncor	TGGATCCTGCTGCTGCTTACCT	GGCTGCTCTCGTGGGGACAGT
Pdk4	GATTGACATCCTGCCTGACC	CATGGAACTCCACCAAATCC
Pltp	ACTACTAAGCTTGGTCGCCAT	CCTGCTTCACCAGATCCAGA
Ppara	AATGCAATTCGCTTTGGAAG	GGCCTTGACCTTGTTCATGT
Scd1	CAGGTTTCCAAGCGCAGTTC	ACTGGAGATCTCTTGGAGCA
Smrt	GCCCTTAGTCCTAGGTGTGG	TTGTACAGAGGCGTGTGGGA
Srebf	GCTTCTCTGGGCTCCTCTCT	TGGCTCCTCTTTGATTCCAG
Tbl1	CGGCGAGGGTGGTCCTGGACTT	CCAGAAAGTTCACCTCGTCGCTGG
Tblr1	TGCCGCCACTAACCAGCAAGG	CATGGCCCCGAAGCACAACCG
Tgfb1	GTCACTGGAGTTGTACGGCA	GGGCTGATCCCGTTGATTTC
Timp1	CAGATACCATGATGGCCCCC	CCCTTATGACCAGGTCCGAG
Tnfa	AGCCCACGTCGTAGCAAACC	GAGGAGCACGTAGTCGGGGC
ChIP Control	GTACCACAGCCTGCACGTAA	ACTGTGCAGCATACCAGTGA
ChIP *Cyp4a14* P	CCATTTCTTGGGACAGGTCA	CGCCAAAGCTCTTTCCACTA
ChIP *Fgf21* P	GGCTTCAGTGTCTTGGTCGT	GTCTTCCTGCTGGGGGTCTA
ChIP *Pdk4* E	TACCACCTTGCTTTCCCAAG	CTTCCCCATGTTGACTGAGC
ChIP *Pdk4* P	GCCACACCAATCAGCTCAGA	GAAACCGTGTCGGGATCACT

Supplementary Table 1: Primers for qPCR and ChIP qPCR.

GPS2 correlated gene list:

PRELP	JUNB	LAPTM5	BSG	CYP1A1	MYO1F
TIMP1	DHCR7	VIM	B2M	ABCD3	NCF2
CTSD	ITGAX	CD68	N4BP2L1	ZFP1	ACLY
TPP1	IL2RG	UBD	GMFG	TTC36	
GRN	CCL21	JUNB	ASPN	PPIB	
KCNN2	DTNA	ALOX5AP	CD53	CCND1	
IGFBP5	FAT1	IGFBP7	IL18	S1PR1	
PRAS	FLNA	AVP	GLIPR1	SERPINA4	
IFITM2	UCP2	FBLN5	WIPI1	GRR65	
HSPB1.2	HLA.DQA1.2	TGFB1	PDGFRA	SCPEP1	
TTYH3	HLA.DQA1.3	PTGIS	TMSB4X	ME1	
SCD	ACTA2	SULF2	MTHFD2L	KPNA2	
MMP2.1	MGP	LTBP4	AASS	TMSB10	
COL1A2	KRT18	HSPB1.1	AOX1	HLA.DRA	

Supplementary Table 2: GPS2 correlated NASH gene list in human subjects.
