# Supplementary material for: Hepatocyte-specific loss of GPS2 in mice reduces non-alcoholic steatohepatitis via activation of PPARα
Source: Nat Commun. 2019 Apr 11;10:1684. doi: 10.1038/s41467-019-09524-z (PMC6459876; doi:10.1038/s41467-019-09524-z)
Supplement: Supplementary file 3 — Reporting Summary [file 41467_2019_9524_MOESM3_ESM.pdf]

nature research

Corresponding author(s): Rongrong Fan & Eckardt Treuter

Last updated by author(s): Feb 27, 2019

Reporting Summary

Nature Research wishes to improve the reproducibility of the work that we publish. This form provides structure for consistency and transparency in reporting. For further information on Nature Research policies, see Authors & Referees and the Editorial Policy Checklist.

Statistics

For all statistical analyses, confirm that the following items are present in the figure legend, table legend, main text, or Methods section.

n/a	Confirmed	
☐	☒	The exact sample size (*n*) for each experimental group/condition, given as a discrete number and unit of measurement
☐	☒	A statement on whether measurements were taken from distinct samples or whether the same sample was measured repeatedly
☐	☒	The statistical test(s) used AND whether they are one- or two-sided<br>*Only common tests should be described solely by name; describe more complex techniques in the Methods section.*
☐	☒	A description of all covariates tested
☐	☒	A description of any assumptions or corrections, such as tests of normality and adjustment for multiple comparisons
☐	☒	A full description of the statistical parameters including central tendency (e.g. means) or other basic estimates (e.g. regression coefficient) AND variation (e.g. standard deviation) or associated estimates of uncertainty (e.g. confidence intervals)
☐	☒	For null hypothesis testing, the test statistic (e.g. *F*, *t*, *r*) with confidence intervals, effect sizes, degrees of freedom and *P* value noted<br>*Give P values as exact values whenever suitable.*
☒	☐	For Bayesian analysis, information on the choice of priors and Markov chain Monte Carlo settings
☒	☐	For hierarchical and complex designs, identification of the appropriate level for tests and full reporting of outcomes
☒	☐	Estimates of effect sizes (e.g. Cohen's *d*, Pearson's *r*), indicating how they were calculated

Our web collection on statistics for biologists contains articles on many of the points above.

Software and code

Policy information about availability of computer code

Data collection	The microarray data from public dataset was imported and analyzed using R packages as stated in the methodology/microarray analysis part. The ChIP-seq data from the public dataset was imported and analyzed using HOMER package and IGV as described in methodology/ChIP-seq data analysis part.
Data analysis	RNA-seq analysis was performed using HISAT2, HOMER and Bioconductor/edgeR packages as described in methodology/RNA-seq part. ChIP-seq analysis was performed using Bowtie2 and HOMER package and viewed in IGV. Cluster 3.0 and TreeView were used to generate and visulize heatmap. Differential peak change was compared using edgeR package.

For manuscripts utilizing custom algorithms or software that are central to the research but not yet described in published literature, software must be made available to editors/reviewers. We strongly encourage code deposition in a community repository (e.g. GitHub). See the Nature Research guidelines for submitting code & software for further information.

Data

Policy information about availability of data

All manuscripts must include a data availability statement. This statement should provide the following information, where applicable:

- Accession codes, unique identifiers, or web links for publicly available datasets
- A list of figures that have associated raw data
- A description of any restrictions on data availability

The datasets generated during and/or analysed during the current study are available in NCBI GEO with access number GSE113157.

Field-specific reporting

Please select the one below that is the best fit for your research. If you are not sure, read the appropriate sections before making your selection.

☐ Life sciences ☐ Behavioural & social sciences ☐ Ecological, evolutionary & environmental sciences

For a reference copy of the document with all sections, see nature.com/documents/nr-reporting-summary-flat.pdf

Life sciences study design

All studies must disclose on these points even when the disclosure is negative.

Sample size	Sample size was not pre-determined. For most of the mice experiments, the sample size is at least 5 in each group. For ChIP-seq and cell experiments, at least biological triplicates were used in repeated experiments.
Data exclusions	Data exclusion is determined by testing the significant outlier using Grubbs' test.
Replication	All the data in the manuscript is at least performed in replicate and repeated at least twice.
Randomization	The animals were selected to ensure 1)they were from the same breedings; 2) they were of same age; and 3) they had similar body weight; and they were then randomly allocated to each experimental group.
Blinding	The experiments were performed by technicians and half blind to the researcher.

Behavioural & social sciences study design

All studies must disclose on these points even when the disclosure is negative.

Study description	*Briefly describe the study type including whether data are quantitative, qualitative, or mixed-methods (e.g. qualitative cross-sectional, quantitative experimental, mixed-methods case study).*
Research sample	*State the research sample (e.g. Harvard university undergraduates, villagers in rural India) and provide relevant demographic information (e.g. age, sex) and indicate whether the sample is representative. Provide a rationale for the study sample chosen. For studies involving existing datasets, please describe the dataset and source.*
Sampling strategy	*Describe the sampling procedure (e.g. random, snowball, stratified, convenience). Describe the statistical methods that were used to predetermine sample size OR if no sample-size calculation was performed, describe how sample sizes were chosen and provide a rationale for why these sample sizes are sufficient. For qualitative data, please indicate whether data saturation was considered, and what criteria were used to decide that no further sampling was needed.*
Data collection	*Provide details about the data collection procedure, including the instruments or devices used to record the data (e.g. pen and paper, computer, eye tracker, video or audio equipment) whether anyone was present besides the participant(s) and the researcher, and whether the researcher was blind to experimental condition and/or the study hypothesis during data collection.*
Timing	*Indicate the start and stop dates of data collection. If there is a gap between collection periods, state the dates for each sample cohort.*
Data exclusions	*If no data were excluded from the analyses, state so OR if data were excluded, provide the exact number of exclusions and the rationale behind them, indicating whether exclusion criteria were pre-established.*
Non-participation	*State how many participants dropped out/declined participation and the reason(s) given OR provide response rate OR state that no participants dropped out/declined participation.*
Randomization	*If participants were not allocated into experimental groups, state so OR describe how participants were allocated to groups, and if allocation was not random, describe how covariates were controlled.*

Ecological, evolutionary & environmental sciences study design

All studies must disclose on these points even when the disclosure is negative.

Study description	*Briefly describe the study. For quantitative data include treatment factors and interactions, design structure (e.g. factorial, nested, hierarchical), nature and number of experimental units and replicates.*
Research sample	*Describe the research sample (e.g. a group of tagged Passer domesticus, all Stenocereus thurberi within Organ Pipe Cactus National Monument), and provide a rationale for the sample choice. When relevant, describe the organism taxa, source, sex, age range and any manipulations. State what population the sample is meant to represent when applicable. For studies involving existing datasets,*

describe the data and its source.

Sampling strategy	*Note the sampling procedure. Describe the statistical methods that were used to predetermine sample size OR if no sample-size calculation was performed, describe how sample sizes were chosen and provide a rationale for why these sample sizes are sufficient.*
Data collection	*Describe the data collection procedure, including who recorded the data and how.*
Timing and spatial scale	*Indicate the start and stop dates of data collection, noting the frequency and periodicity of sampling and providing a rationale for these choices. If there is a gap between collection periods, state the dates for each sample cohort. Specify the spatial scale from which the data are taken*
Data exclusions	*If no data were excluded from the analyses, state so OR if data were excluded, describe the exclusions and the rationale behind them, indicating whether exclusion criteria were pre-established.*
Reproducibility	*Describe the measures taken to verify the reproducibility of experimental findings. For each experiment, note whether any attempts to repeat the experiment failed OR state that all attempts to repeat the experiment were successful.*
Randomization	*Describe how samples/organisms/participants were allocated into groups. If allocation was not random, describe how covariates were controlled. If this is not relevant to your study, explain why.*
Blinding	*Describe the extent of blinding used during data acquisition and analysis. If blinding was not possible, describe why OR explain why blinding was not relevant to your study.*

Did the study involve field work? ☐ Yes ☐ No

Field work, collection and transport

Field conditions	*Describe the study conditions for field work, providing relevant parameters (e.g. temperature, rainfall).*
Location	*State the location of the sampling or experiment, providing relevant parameters (e.g. latitude and longitude, elevation, water depth).*
Access and import/export	*Describe the efforts you have made to access habitats and to collect and import/export your samples in a responsible manner and in compliance with local, national and international laws, noting any permits that were obtained (give the name of the issuing authority, the date of issue, and any identifying information).*
Disturbance	*Describe any disturbance caused by the study and how it was minimized.*

Reporting for specific materials, systems and methods

We require information from authors about some types of materials, experimental systems and methods used in many studies. Here, indicate whether each material, system or method listed is relevant to your study. If you are not sure if a list item applies to your research, read the appropriate section before selecting a response.

Materials & experimental systems

n/a	Involved in the study	
☐	☒	Antibodies
☐	☒	Eukaryotic cell lines
☐	☐	Palaeontology
☐	☒	Animals and other organisms
☐	☐	Human research participants
☐	☐	Clinical data

Methods

n/a	Involved in the study	
☐	☒	ChIP-seq
☐	☐	Flow cytometry
☐	☐	MRI-based neuroimaging

Antibodies

Antibodies used	All the antibodies were described in detail in methodology/ChIP and ChIP-seq sample preparation; and methodology/western blot.
Validation	The key antibodies used in this manuscript (PPARa and GPS2) were validated using KO mouse tissues. The rest of the antibodies were based on previous published literatures. All the detailed information of the antibody has been described in the methodology part.

Eukaryotic cell lines

Policy information about cell lines

Cell line source(s)	All the cell lines used in the manuscript are from ATCC as described in methodology/cell culture studies.
Authentication	The AML12 cells were authenticated by measuring the expression of Albumin, HK293 cells were not authenticated.
Mycoplasma contamination	All the cells have been tested for mycoplasma contaminations.
Commonly misidentified lines (See ICLAC register)	*Name any commonly misidentified cell lines used in the study and provide a rationale for their use.*

Palaeontology

Specimen provenance	*Provide provenance information for specimens and describe permits that were obtained for the work (including the name of the issuing authority, the date of issue, and any identifying information).*
Specimen deposition	*Indicate where the specimens have been deposited to permit free access by other researchers.*
Dating methods	*If new dates are provided, describe how they were obtained (e.g. collection, storage, sample pretreatment and measurement), where they were obtained (i.e. lab name), the calibration program and the protocol for quality assurance OR state that no new dates are provided.*

☐ Tick this box to confirm that the raw and calibrated dates are available in the paper or in Supplementary Information.

Animals and other organisms

Policy information about studies involving animals; ARRIVE guidelines recommended for reporting animal research

Laboratory animals	The laboratory animals used in this study have been carefully described in methodology/animals part.
Wild animals	*Provide details on animals observed in or captured in the field; report species, sex and age where possible. Describe how animals were caught and transported and what happened to captive animals after the study (if killed, explain why and describe method; if released, say where and when) OR state that the study did not involve wild animals.*
Field-collected samples	*For laboratory work with field-collected samples, describe all relevant parameters such as housing, maintenance, temperature, photoperiod and end-of-experiment protocol OR state that the study did not involve samples collected from the field.*
Ethics oversight	All animal experiments were approved by the respective national ethical boards (Swedish Board of Agriculture, Stockholm South, S28-12, S30-14, S135-12, S29-14, ID907)

Note that full information on the approval of the study protocol must also be provided in the manuscript.

Human research participants

Policy information about studies involving human research participants

Population characteristics	*Describe the covariate-relevant population characteristics of the human research participants (e.g. age, gender, genotypic information, past and current diagnosis and treatment categories). If you filled out the behavioural & social sciences study design questions and have nothing to add here, write "See above."*
Recruitment	*Describe how participants were recruited. Outline any potential self-selection bias or other biases that may be present and how these are likely to impact results.*
Ethics oversight	*Identify the organization(s) that approved the study protocol.*

Note that full information on the approval of the study protocol must also be provided in the manuscript.

Clinical data

Policy information about clinical studies

All manuscripts should comply with the ICMJE guidelines for publication of clinical research and a completed CONSORT checklist must be included with all submissions.

Clinical trial registration	The clinical data is obtained through published online resources.
Study protocol	*Note where the full trial protocol can be accessed OR if not available, explain why.*
Data collection	*Describe the settings and locales of data collection, noting the time periods of recruitment and data collection.*

Outcomes

Describe how you pre-defined primary and secondary outcome measures and how you assessed these measures.

ChIP-seq

Data deposition

☒ Confirm that both raw and final processed data have been deposited in a public database such as GEO.

☒ Confirm that you have deposited or provided access to graph files (e.g. BED files) for the called peaks.

Data access links
May remain private before publication.

All sequencing data in this study are available in the GEO under the accession number GSE113157.
https://www.ncbi.nlm.nih.gov/geo/query/acc.cgi?acc=GSE113157

Files in database submission

The table of all files in database submission is given below.
GPS2_ChIPseq_GPS2_LKO.fastq.gz
GPS2_ChIPseq_GPS2_WT.fastq.gz
GPS2_ChIPseq_NCOR_LKO_1.fastq.gz
GPS2_ChIPseq_NCOR_LKO_2.fastq.gz
GPS2_ChIPseq_NCOR_LKO_3.fastq.gz
GPS2_ChIPseq_NCOR_WT_1.fastq.gz
GPS2_ChIPseq_NCOR_WT_2.fastq.gz
GPS2_ChIPseq_NCOR_WT_3.fastq.gz
GPS2_ChIPseq_PPARa_KO_1.fastq.gz
GPS2_ChIPseq_PPARa_KO_2.fastq.gz
GPS2_ChIPseq_PPARa_KO_3.fastq.gz
GPS2_ChIPseq_PPARa_WT_1.fastq.gz
GPS2_ChIPseq_PPARa_WT_2.fastq.gz
GPS2_ChIPseq_PPARa_WT_3.fastq.gz
H3K27ac_ChIPseq_GPS2_LKO_1.txt.gz
H3K27ac_ChIPseq_GPS2_LKO_2.txt.gz
H3K27ac_ChIPseq_GPS2_WT_1.txt.gz
H3K27ac_ChIPseq_GPS2_WT_2.txt.gz
H3K27ac_ChIPseq_NCOR_LKO_1.fastq.gz
H3K27ac_ChIPseq_NCOR_LKO_2.fastq.gz
H3K27ac_ChIPseq_NCOR_LKO_3.fastq.gz
H3K27ac_ChIPseq_NCOR_WT_1.fastq.gz
H3K27ac_ChIPseq_NCOR_WT_2.fastq.gz
H3K27ac_ChIPseq_NCOR_WT_3.fastq.gz
H3K27ac_ChIPseq_SMRT_LKO_1.fastq.gz
H3K27ac_ChIPseq_SMRT_LKO_2.fastq.gz
H3K27ac_ChIPseq_SMRT_LKO_3.fastq.gz
H3K27ac_ChIPseq_SMRT_WT_1.fastq.gz
H3K27ac_ChIPseq_SMRT_WT_2.fastq.gz
H3K27ac_ChIPseq_SMRT_WT_3.fastq.gz
H3K4me3_ChIPseq_GPS2_LKO_1.txt.gz
H3K4me3_ChIPseq_GPS2_LKO_2.txt.gz
H3K4me3_ChIPseq_GPS2_WT_1.txt.gz
H3K4me3_ChIPseq_GPS2_WT_2.txt.gz
NCOR_ChIPseq_GPS2_LKO_1.fastq.gz
NCOR_ChIPseq_GPS2_LKO_2.fastq.gz
NCOR_ChIPseq_GPS2_LKO_3.fastq.gz
NCOR_ChIPseq_GPS2_WT_1.fastq.gz
NCOR_ChIPseq_GPS2_WT_2.fastq.gz
NCOR_ChIPseq_GPS2_WT_3.fastq.gz
NCOR_ChIPseq_PPARa_KO_1.fastq.gz
NCOR_ChIPseq_PPARa_KO_2.fastq.gz
NCOR_ChIPseq_PPARa_KO_3.fastq.gz
NCOR_ChIPseq_PPARa_WT_1.fastq.gz
NCOR_ChIPseq_PPARa_WT_2.fastq.gz
NCOR_ChIPseq_PPARa_WT_3.fastq.gz
PolII_ChIPseq_GPS2_LKO.fastq.gz
PolII_ChIPseq_GPS2_WT.fastq.gz
PPARa_ChIPseq_GPS2_LKO_1.fastq.gz
PPARa_ChIPseq_GPS2_LKO_2.fastq.gz
PPARa_ChIPseq_GPS2_LKO_3.fastq.gz
PPARa_ChIPseq_GPS2_WT_1.fastq.gz
PPARa_ChIPseq_GPS2_WT_2.fastq.gz
PPARa_ChIPseq_GPS2_WT_3.fastq.gz
PPARa_ChIPseq_NCOR_LKO_1.fastq.gz
PPARa_ChIPseq_NCOR_LKO_2.fastq.gz
PPARa_ChIPseq_NCOR_LKO_3.fastq.gz
PPARa_ChIPseq_NCOR_WT_1.fastq.gz
PPARa_ChIPseq_NCOR_WT_2.fastq.gz

PPARa_ChIPseq_NCOR_WT_3.fastq.gz
PPARa_ChIPseq_PPARa_KO_1.fastq.gz
PPARa_ChIPseq_PPARa_KO_2.fastq.gz
PPARa_ChIPseq_PPARa_WT_1.fastq.gz
PPARa_ChIPseq_PPARa_WT_2.fastq.gz
raw_tag_counts_of_GPS2_peaks_in_GPS2_WT_LKO_livers.txt
raw_tag_counts_of_GPS2_peaks_in_NCOR_WT_LKO_livers.txt
raw_tag_counts_of_GPS2_peaks_in_PPARa_WT_KO_livers.txt
raw_tag_counts_of_H3K27ac_peaks_in_NCOR_SMRT_WT_LKO_livers.txt
raw_tag_counts_of_H3K4me3_H3K27ac_peaks_in_GPS2_WT_LKO_livers.txt
raw_tag_counts_of_NCOR_peaks_in_GPS2_WT_LKO_livers.txt
raw_tag_counts_of_NCOR_peaks_in_PPARa_WT_KO_livers.txt
raw_tag_counts_of_PolII_peaks_in_GPS2_WT_LKO_livers.txt
raw_tag_counts_of_PPARa_peaks_in_GPS2_WT_LKO_livers.txt
raw_tag_counts_of_PPARa_peaks_in_NCOR_WT_LKO_livers.txt
raw_tag_counts_of_PPARa_peaks_in_PPARa_WT_KO_livers.txt
GPS2_ChIPseq_GPS2_LKO.bed
GPS2_ChIPseq_GPS2_WT.bed
GPS2_ChIPseq_NCOR_LKO_1.bed
GPS2_ChIPseq_NCOR_LKO_2.bed
GPS2_ChIPseq_NCOR_LKO_3.bed
GPS2_ChIPseq_NCOR_WT_1.bed
GPS2_ChIPseq_NCOR_WT_2.bed
GPS2_ChIPseq_NCOR_WT_3.bed
GPS2_ChIPseq_PPARa_KO_1.bed
GPS2_ChIPseq_PPARa_KO_2.bed
GPS2_ChIPseq_PPARa_KO_3.bed
GPS2_ChIPseq_PPARa_WT_1.bed
GPS2_ChIPseq_PPARa_WT_2.bed
GPS2_ChIPseq_PPARa_WT_3.bed
H3K4me3_ChIPseq_GPS2_LKO_1.bed
H3K4me3_ChIPseq_GPS2_LKO_2.bed
H3K4me3_ChIPseq_GPS2_WT_1.bed
H3K4me3_ChIPseq_GPS2_WT_2.bed
H3K27ac_ChIPseq_GPS2_LKO_1.bed
H3K27ac_ChIPseq_GPS2_LKO_2.bed
H3K27ac_ChIPseq_GPS2_WT_1.bed
H3K27ac_ChIPseq_GPS2_WT_2.bed
H3K27ac_ChIPseq_NCOR_LKO_1.bed
H3K27ac_ChIPseq_NCOR_LKO_2.bed
H3K27ac_ChIPseq_NCOR_LKO_3.bed
H3K27ac_ChIPseq_NCOR_WT_1.bed
H3K27ac_ChIPseq_NCOR_WT_2.bed
H3K27ac_ChIPseq_NCOR_WT_3.bed
H3K27ac_ChIPseq_SMRT_LKO_1.bed
H3K27ac_ChIPseq_SMRT_LKO_2.bed
H3K27ac_ChIPseq_SMRT_LKO_3.bed
H3K27ac_ChIPseq_SMRT_WT_1.bed
H3K27ac_ChIPseq_SMRT_WT_2.bed
H3K27ac_ChIPseq_SMRT_WT_3.bed
NCOR_ChIPseq_GPS2_LKO_1.bed
NCOR_ChIPseq_GPS2_LKO_2.bed
NCOR_ChIPseq_GPS2_LKO_3.bed
NCOR_ChIPseq_GPS2_WT_1.bed
NCOR_ChIPseq_GPS2_WT_2.bed
NCOR_ChIPseq_GPS2_WT_3.bed
NCOR_ChIPseq_PPARa_KO_1.bed
NCOR_ChIPseq_PPARa_KO_2.bed
NCOR_ChIPseq_PPARa_KO_3.bed
NCOR_ChIPseq_PPARa_WT_1.bed
NCOR_ChIPseq_PPARa_WT_2.bed
NCOR_ChIPseq_PPARa_WT_3.bed
PolII_ChIPseq_GPS2_LKO.bed
PolII_ChIPseq_GPS2_WT.bed
PPARa_ChIPseq_GPS2_LKO_1.bed
PPARa_ChIPseq_GPS2_LKO_2.bed
PPARa_ChIPseq_GPS2_LKO_3.bed
PPARa_ChIPseq_GPS2_WT_1.bed
PPARa_ChIPseq_GPS2_WT_2.bed
PPARa_ChIPseq_GPS2_WT_3.bed
PPARa_ChIPseq_NCOR_LKO_1.bed
PPARa_ChIPseq_NCOR_LKO_2.bed
PPARa_ChIPseq_NCOR_LKO_3.bed
PPARa_ChIPseq_NCOR_WT_1.bed
PPARa_ChIPseq_NCOR_WT_2.bed

PPARa_ChIPseq_NCOR_WT_3.bed
PPARa_ChIPseq_PPARa_KO_1.bed
PPARa_ChIPseq_PPARa_KO_2.bed
PPARa_ChIPseq_PPARa_WT_1.bed
PPARa_ChIPseq_PPARa_WT_2.bed

Genome browser session (e.g. UCSC)

no longer applicable

Methodology

Replicates

ChIP-seq sample replicate(s) type
PolII_ChIPseq_in_GPS2_WT 1 biological
PolII_ChIPseq_in_GPS2_LKO 1 biological
GPS2_ChIPseq_in_GPS2_WT 1 biological
GPS2_ChIPseq_in_GPS2_LKO 1 biological
GPS2_ChIPseq_in_NCOR_WT 3 biological
GPS2_ChIPseq_in_NCOR_LKO 3 biological
GPS2_ChIPseq_in_PPARa_WT 3 biological
GPS2_ChIPseq_in_PPARa_KO 3 biological
NCOR_ChIPseq_in_GPS2_WT 3 biological
NCOR_ChIPseq_in_GPS2_LKO 3 biological
NCOR_ChIPseq_in_PPARa_WT 3 biological
NCOR_ChIPseq_in_PPARa_KO 3 biological
PPARa_ChIPseq_in_GPS2_WT 3 biological
PPARa_ChIPseq_in_GPS2_LKO 3 biological
PPARa_ChIPseq_in_NCOR_WT 3 biological
PPARa_ChIPseq_in_NCOR_LKO 3 biological
PPARa_ChIPseq_in_PPARa_WT 2 biological
PPARa_ChIPseq_in_PPARa_KO 2 biological
H3K27ac_ChIPseq_in_GPS2_WT 2 biological
H3K27ac_ChIPseq_in_GPS2_LKO 2 biological
H3K27ac_ChIPseq_in_NCOR_WT 3 biological
H3K27ac_ChIPseq_in_NCOR_LKO 3 biological
H3K27ac_ChIPseq_in_SMRT_WT 3 biological
H3K27ac_ChIPseq_in_SMRT_LKO 3 biological
H3K4me3_ChIPseq_in_GPS2_WT 2 biological
H3K4me3_ChIPseq_in_GPS2_LKO 2 biological

Sequencing depth

All ChIP-seq samples are sequenced in single-end.
ChIPseq samples total reads unique reads length of the reads
GPS2_ChIPseq_GPS2_LKO 21971756 14849353 50
GPS2_ChIPseq_GPS2_WT 23989865 16112504 75
GPS2_ChIPseq_NCOR_LKO_1 33531452 22770199 75
GPS2_ChIPseq_NCOR_LKO_2 29769462 19942894 75
GPS2_ChIPseq_NCOR_LKO_3 30771608 20653723 75
GPS2_ChIPseq_NCOR_WT_1 26122746 18262084 75
GPS2_ChIPseq_NCOR_WT_2 25189572 16976316 75
GPS2_ChIPseq_NCOR_WT_3 32430711 21395039 75
GPS2_ChIPseq_PPARa_KO_1 22453999 13841110 75
GPS2_ChIPseq_PPARa_KO_2 21563089 13243893 75
GPS2_ChIPseq_PPARa_KO_3 21943821 13675915 75
GPS2_ChIPseq_PPARa_WT_1 23078492 14392864 75
GPS2_ChIPseq_PPARa_WT_2 24169730 15330977 75
GPS2_ChIPseq_PPARa_WT_3 20753916 13374049 75
H3K27ac_ChIPseq_GPS2_LKO_1 33323021 29758416 50
H3K27ac_ChIPseq_GPS2_LKO_2 38315563 33915676 50
H3K27ac_ChIPseq_GPS2_WT_1 31398297 27923028 50
H3K27ac_ChIPseq_GPS2_WT_2 39138126 34530148 50
H3K27ac_ChIPseq_NCOR_LKO_1 65874134 57724627 75
H3K27ac_ChIPseq_NCOR_LKO_2 57419281 49954427 75
H3K27ac_ChIPseq_NCOR_LKO_3 65906508 57297497 75
H3K27ac_ChIPseq_NCOR_WT_1 53116928 46186647 75
H3K27ac_ChIPseq_NCOR_WT_2 60238382 52826609 75
H3K27ac_ChIPseq_NCOR_WT_3 65546494 56827241 75
H3K27ac_ChIPseq_SMRT_LKO_1 50402593 44007532 75
H3K27ac_ChIPseq_SMRT_LKO_2 56126071 48900736 75
H3K27ac_ChIPseq_SMRT_LKO_3 49485346 42945110 75
H3K27ac_ChIPseq_SMRT_WT_1 49597001 43357575 75
H3K27ac_ChIPseq_SMRT_WT_2 61257770 53722391 75
H3K27ac_ChIPseq_SMRT_WT_3 49540723 43017985 75
H3K4me3_ChIPseq_GPS2_LKO_1 31142402 23867145 50
H3K4me3_ChIPseq_GPS2_LKO_2 30758669 23918636 50
H3K4me3_ChIPseq_GPS2_WT_1 28120273 20873899 50
H3K4me3_ChIPseq_GPS2_WT_2 23789764 17986365 50

NCOR_ChIPseq_GPS2_LKO_1 31132349 21508926 75
NCOR_ChIPseq_GPS2_LKO_2 27853623 19775241 75
NCOR_ChIPseq_GPS2_LKO_3 28034005 18485370 75
NCOR_ChIPseq_GPS2_WT_1 31747477 22431351 75
NCOR_ChIPseq_GPS2_WT_2 26905182 18583426 75
NCOR_ChIPseq_GPS2_WT_3 25758125 17227949 75
NCOR_ChIPseq_PPARa_KO_1 20072491 13561949 75
NCOR_ChIPseq_PPARa_KO_2 24267838 16581490 75
NCOR_ChIPseq_PPARa_KO_3 22289899 14847628 75
NCOR_ChIPseq_PPARa_WT_1 20307552 13848031 75
NCOR_ChIPseq_PPARa_WT_2 19260186 13423208 75
NCOR_ChIPseq_PPARa_WT_3 19606333 13911183 75
PolII_ChIPseq_GPS2_LKO 22012062 14599460 75
PolII_ChIPseq_GPS2_WT 20471305 13783056 75
PPARa_ChIPseq_GPS2_LKO_1 12953729 9592984 75
PPARa_ChIPseq_GPS2_LKO_2 15864183 11682949 75
PPARa_ChIPseq_GPS2_LKO_3 26196036 19404694 75
PPARa_ChIPseq_GPS2_WT_1 12469647 11682949 75
PPARa_ChIPseq_GPS2_WT_2 16811334 11682949 75
PPARa_ChIPseq_GPS2_WT_3 13993200 9488668 75
PPARa_ChIPseq_NCOR_LKO_1 27648174 18327775 75
PPARa_ChIPseq_NCOR_LKO_2 29092662 19486002 75
PPARa_ChIPseq_NCOR_LKO_3 25897944 17762645 75
PPARa_ChIPseq_NCOR_WT_1 24881313 17139100 75
PPARa_ChIPseq_NCOR_WT_2 28797847 19852116 75
PPARa_ChIPseq_NCOR_WT_3 33589314 21567014 75
PPARa_ChIPseq_PPARa_KO_1 23806649 14593782 75
PPARa_ChIPseq_PPARa_KO_2 22586598 13642031 75
PPARa_ChIPseq_PPARa_WT_1 22550300 13986044 75
PPARa_ChIPseq_PPARa_WT_2 20607127 12963222 75

Antibodies

PPARa (Millipore, MAB3890)
PolII (BioLegend, 664906)
NCOR (Bethyl laboratories, A301-145A)
H3K4me3 (Abcam, ab8580)
H3K27ac (Abcam, ab4729)
GPS2(Home-made)

Peak calling parameters

Sequenced raw data (in fastq) were aligned to mouse mm9 genome using Bowtie2 program with all default settings.

All peaks were determined by the HOMER findPeaks program against the input samples with the following options:
-i <input tag directory> (input sample)
-tbp 1 (maximum 1 tag per bp to count);
-inputtbp 1 (maximum 1 tag per bp to count in Input);
-style histone (for H3K4me3 and H3K27ac ChIPseq) or -style factor (for GPS2 NCOR PPARa PolII ChIPseq)
other default settings:
-gsize (Set effective mappable genome size, default: 2e9)
-F (fold enrichment over input tag count, default: 4.0);
-L (fold enrichment over local tag count, default: 4.0);
-C (fold enrichment limit of expected unique tag positions, default: 2.0);
-fdr (False discovery rate, default = 0.001)

Data quality

As described above, we only select the peaks with more than 4 fold change over local tag counts as well as input tag counts while the FDR less than 0.001. Still we got around 30k-80k peaks from most of the experiments.
ChIPseq samples peak number
GPS2_ChIPseq_GPS2_LKO 52
GPS2_ChIPseq_GPS2_WT 48968
GPS2_ChIPseq_NCOR_LKO_1 13260
GPS2_ChIPseq_NCOR_LKO_2 14182
GPS2_ChIPseq_NCOR_LKO_3 9563
GPS2_ChIPseq_NCOR_WT_1 46157
GPS2_ChIPseq_NCOR_WT_2 34941
GPS2_ChIPseq_NCOR_WT_3 13863
GPS2_ChIPseq_PPARa_KO_1 7479
GPS2_ChIPseq_PPARa_KO_2 5450
GPS2_ChIPseq_PPARa_KO_3 806
GPS2_ChIPseq_PPARa_WT_1 11339
GPS2_ChIPseq_PPARa_WT_2 11572
GPS2_ChIPseq_PPARa_WT_3 20818
H3K27ac_ChIPseq_GPS2_LKO_1 51462
H3K27ac_ChIPseq_GPS2_LKO_2 51281
H3K27ac_ChIPseq_GPS2_WT_1 50269
H3K27ac_ChIPseq_GPS2_WT_2 49995
H3K27ac_ChIPseq_NCOR_LKO_1 47202
H3K27ac_ChIPseq_NCOR_LKO_2 50568

H3K27ac_ChIPseq_NCOR_LKO_3 50616
H3K27ac_ChIPseq_NCOR_WT_1 50067
H3K27ac_ChIPseq_NCOR_WT_2 48243
H3K27ac_ChIPseq_NCOR_WT_3 48751
H3K27ac_ChIPseq_SMRT_LKO_1 50224
H3K27ac_ChIPseq_SMRT_LKO_2 48705
H3K27ac_ChIPseq_SMRT_LKO_3 49017
H3K27ac_ChIPseq_SMRT_WT_1 47716
H3K27ac_ChIPseq_SMRT_WT_2 48126
H3K27ac_ChIPseq_SMRT_WT_3 48128
H3K4me3_ChIPseq_GPS2_LKO_1 24770
H3K4me3_ChIPseq_GPS2_LKO_2 23768
H3K4me3_ChIPseq_GPS2_WT_1 25542
H3K4me3_ChIPseq_GPS2_WT_2 23782
NCOR_ChIPseq_GPS2_LKO_1 47656
NCOR_ChIPseq_GPS2_LKO_2 54329
NCOR_ChIPseq_GPS2_LKO_3 30893
NCOR_ChIPseq_GPS2_WT_1 53145
NCOR_ChIPseq_GPS2_WT_2 50151
NCOR_ChIPseq_GPS2_WT_3 31561
NCOR_ChIPseq_PPARa_KO_1 69983
NCOR_ChIPseq_PPARa_KO_2 75093
NCOR_ChIPseq_PPARa_KO_3 58459
NCOR_ChIPseq_PPARa_WT_1 78279
NCOR_ChIPseq_PPARa_WT_2 75828
NCOR_ChIPseq_PPARa_WT_3 79344
PolII_ChIPseq_GPS2_LKO 26420
PolII_ChIPseq_GPS2_WT 25825
PPARa_ChIPseq_GPS2_LKO_1 68891
PPARa_ChIPseq_GPS2_LKO_2 72003
PPARa_ChIPseq_GPS2_LKO_3 79777
PPARa_ChIPseq_GPS2_WT_1 47353
PPARa_ChIPseq_GPS2_WT_2 61659
PPARa_ChIPseq_GPS2_WT_3 51934
PPARa_ChIPseq_NCOR_LKO_1 55059
PPARa_ChIPseq_NCOR_LKO_2 55389
PPARa_ChIPseq_NCOR_LKO_3 63975
PPARa_ChIPseq_NCOR_WT_1 61867
PPARa_ChIPseq_NCOR_WT_2 61375
PPARa_ChIPseq_NCOR_WT_3 40057
PPARa_ChIPseq_PPARa_KO_1 131
PPARa_ChIPseq_PPARa_KO_2 92
PPARa_ChIPseq_PPARa_WT_1 8975
PPARa_ChIPseq_PPARa_WT_2 1274

Software

Homer(v4.10); Bowtie2 (v2.3.4.3); Cluster (v3.0); TreeView (v3.0)

Flow Cytometry

Plots

Confirm that:

☐ The axis labels state the marker and fluorochrome used (e.g. CD4-FITC).

☐ The axis scales are clearly visible. Include numbers along axes only for bottom left plot of group (a 'group' is an analysis of identical markers).

☐ All plots are contour plots with outliers or pseudocolor plots.

☐ A numerical value for number of cells or percentage (with statistics) is provided.

Methodology

Sample preparation

Describe the sample preparation, detailing the biological source of the cells and any tissue processing steps used.

Instrument

Identify the instrument used for data collection, specifying make and model number.

Software

Describe the software used to collect and analyze the flow cytometry data. For custom code that has been deposited into a community repository, provide accession details.

Cell population abundance

Describe the abundance of the relevant cell populations within post-sort fractions, providing details on the purity of the samples and how it was determined.

Gating strategy | *Describe the gating strategy used for all relevant experiments, specifying the preliminary FSC/SSC gates of the starting cell population, indicating where boundaries between "positive" and "negative" staining cell populations are defined.*

☐ Tick this box to confirm that a figure exemplifying the gating strategy is provided in the Supplementary Information.

Magnetic resonance imaging

Experimental design

Design type	*Indicate task or resting state; event-related or block design.*
Design specifications	*Specify the number of blocks, trials or experimental units per session and/or subject, and specify the length of each trial or block (if trials are blocked) and interval between trials.*
Behavioral performance measures	*State number and/or type of variables recorded (e.g. correct button press, response time) and what statistics were used to establish that the subjects were performing the task as expected (e.g. mean, range, and/or standard deviation across subjects).*

Acquisition

Imaging type(s)	*Specify: functional, structural, diffusion, perfusion.*
Field strength	*Specify in Tesla*
Sequence & imaging parameters	*Specify the pulse sequence type (gradient echo, spin echo, etc.), imaging type (EPI, spiral, etc.), field of view, matrix size, slice thickness, orientation and TE/TR/flip angle.*
Area of acquisition	*State whether a whole brain scan was used OR define the area of acquisition, describing how the region was determined.*

Diffusion MRI ☐ Used ☐ Not used

Preprocessing

Preprocessing software	*Provide detail on software version and revision number and on specific parameters (model/functions, brain extraction, segmentation, smoothing kernel size, etc.).*
Normalization	*If data were normalized/standardized, describe the approach(es): specify linear or non-linear and define image types used for transformation OR indicate that data were not normalized and explain rationale for lack of normalization.*
Normalization template	*Describe the template used for normalization/transformation, specifying subject space or group standardized space (e.g. original Talairach, MNI305, ICBM152) OR indicate that the data were not normalized.*
Noise and artifact removal	*Describe your procedure(s) for artifact and structured noise removal, specifying motion parameters, tissue signals and physiological signals (heart rate, respiration).*
Volume censoring	*Define your software and/or method and criteria for volume censoring, and state the extent of such censoring.*

Statistical modeling & inference

Model type and settings	*Specify type (mass univariate, multivariate, RSA, predictive, etc.) and describe essential details of the model at the first and second levels (e.g. fixed, random or mixed effects; drift or auto-correlation).*
Effect(s) tested	*Define precise effect in terms of the task or stimulus conditions instead of psychological concepts and indicate whether ANOVA or factorial designs were used.*

Specify type of analysis: ☐ Whole brain ☐ ROI-based ☐ Both

Statistic type for inference<br>(See Eklund et al. 2016)	*Specify voxel-wise or cluster-wise and report all relevant parameters for cluster-wise methods.*
Correction	*Describe the type of correction and how it is obtained for multiple comparisons (e.g. FWE, FDR, permutation or Monte Carlo).*

Models & analysis

n/a	Involved in the study
☐	☐ Functional and/or effective connectivity
☐	☐ Graph analysis
☐	☐ Multivariate modeling or predictive analysis

Functional and/or effective connectivity	*Report the measures of dependence used and the model details (e.g. Pearson correlation, partial correlation, mutual information).*
Graph analysis	*Report the dependent variable and connectivity measure, specifying weighted graph or binarized graph, subject- or group-level, and the global and/or node summaries used (e.g. clustering coefficient, efficiency, etc.).*
Multivariate modeling and predictive analysis	*Specify independent variables, features extraction and dimension reduction, model, training and evaluation metrics.*
